# Is surgical intervention more effective than non-surgical treatment for carpal tunnel syndrome? a systematic review

**DOI:** 10.1186/1749-799X-6-17

**Published:** 2011-04-11

**Authors:** Qiyun Shi, Joy C MacDermid

**Affiliations:** 1 Department of Clinical Epidemiology and Biostatistics, McMaster University, Hamilton, Ontario, L8S 4L8, Canada; 2 Hand and Upper Limb Centre Clinical Research Laboratory, St. Joseph's Health Centre, 268 Grosvenor St., London, Ontario, N6A 3A8, Canada; 3 Professor, Assistant Dean of Rehabilitation Science, McMaster University, Hamilton, Ontario, L8S 4L8, Canada

## Abstract

**Background:**

Carpal tunnel syndrome is a common disorder in hand surgery practice. Both surgical and conservative interventions are utilized for the carpal tunnel syndrome. Although certain indications would specifically indicate the need for surgery, there is a spectrum of patients for whom either treatment option might be selected. The purpose of this systematic review was to compare the efficacy of surgical treatment of carpal tunnel syndrome with conservative treatment

**Methods:**

We included all controlled trials written in English, attempting to compare any surgical interventions with any conservative therapies. We searched Cochrane Central Register of Controlled Trials (The Cochrane Library Issue 1, 2010), MEDLINE (1980 to June 2010), EMBASE (1980 to June 2010), PEDro (searched in June 2010), international guidelines, computer searches based on key words and reference lists of articles. Two reviewers performed study selection, assessment of methodological quality and data extraction independently of each other. Weighted mean differences and 95% confidence intervals for patient self-reported functional and symptom questionnaires were calculated. Relative risk (RR) and 95% confidence intervals for electrophysiological studies and complication were also calculated.

**Results:**

We assessed seven studies in this review including 5 RCTs and 2 controlled trials. The methodological quality of the trials ranged from moderate to high. The weighted mean difference demonstrated a larger treatment benefit for surgical intervention compared to non surgical intervention at six months for functional status 0.35( 95% CI 0.22, 0.47) and symptom severity 0.43 (95% CI 0.29, 0.57). There were no statistically significant difference between the intervention options at 3 months but there was a benefit in favor of surgery in terms of function and symptom relief at 12 months ( 0.35, 95% CI 0.15, 0.55 and 0.37, 95% CI 0.19 to 0.56). The RR for secondary outcomes of normal nerve conduction studies was 2.3 (95% CI 1.2, 4.4), while RR was 2.03 (95% CI 1.28 to 3.22) for complication, both favoring surgery.

**Conclusion:**

Both surgical and conservative interventions had treatment benefit in carpal tunnel syndrome. Surgical treatment has a superior benefit, in symptoms and function, at six and twelve months. Patient underwent surgical release were two times more likely to have normal nerve conduction studies but also had complication and side effects as well. Given the treatment differential and potential for adverse effects and that conservative interventions benefitted a substantial proportion of patients, current practice of a trial of conservative management with surgical release for severe or persistent symptoms is supported by evidence.

## Background

Carpal tunnel syndrome (CTS) is the most common entrapment neuropathy [1] in America. The prevalence of CTS is from 1% to 3% [2,3]; with an incidence that peaks in the late 50s [4]. There is a high rate of CTS within certain occupational groups such as meatpackers, poultry processors and automobile assembly workers [5] which is attributed to job tasks that require intensive manual exertion. In addition, CTS is associated with some systemic conditions, such as rheumatoid arthritis, hypothyroidism, diabetes mellitus, gout, and pregnancy [6]. Both conservative and surgical treatments are used to manage CTS. The non-surgical treatment options include splinting, steroids, activity modification, non-steroidal anti-inflammatory drugs, diuretics, vitamin B-6 and others. However, of the conservative approaches only splinting [7] and steroids [8] are supported by high quality evidence.

Surgical release of the carpal tunnel is known to be effective and is typically used for patients who fail to achieve adequate relief with conservative managements and for those with moderate to severe symptoms [9]. Although surgical intervention is considered as the definitive treatment to the CTS, it is not considered a first line of treatment. Conservative intervention may not be curative; but may provide sufficient relief in a proportion of cases. It may also be a patient preference due to concerns about the discomfort, inconvenience or safety of surgery. Conservative management is typically preferred for transient cases of CTS such as those associated with pregnancy or short-term overuse. In other cases conservative management might be used for partial relief of symptoms while awaiting surgery or for diagnostic purposes in determining patient response. Despite, potential variations in indications for one treatment and the associated expectations, there are a substantial proportion of patients for whom conservative management may have provided incomplete relief. These patients require evidence that surgical intervention has is more effective to proceed to surgery.

Systematic reviews provided the best evidence. In 2008, Verdugo et al. [7] conducted a systematic review comparing surgical and non-surgical treatment for CTS; were able to locate four randomized controlled trials. The objective of this study was to build on this work by adopting boarder inclusion criterion, locating more recent trials that conducting a meta-analysis to synthesize evidence in a more quantitative manner.

## Methods

### Literature search

A literature search of four databases was conducted in June 2010 for studies addressing effectiveness of surgical or conservative interventions for CTS. The research strategy is list in Additional File 1.

These databases were Cochrane Central Register of Controlled Trials (CENTRAL) (The Cochrane Library Issue 1, 2010), MEDLINE (1980 to June 2010), EMBASE (1980 to June 2010), PEDro (searched in June 2010). Only English language papers were included. Searching of international guidelines, computer searches based on key words, and hand searching for references from previously retrieved articles was used to extend the search strategy.

Research articles were included for review if they met the following criteria:

1. The study was written in English.

2. The study was designed as a prospective controlled trial.

3. The study subjects/patients had a diagnosis of CTS, irrespective of the diagnostic criteria used, etiology of the syndrome, associated pathology, gender and age.

4. The study compared any surgical with non-surgical intervention.

The surgical treatments include:

1. Standard open carpal tunnel release (OCTR).

2. Endoscopic carpal tunnel release (ECTR).

3. Open carpal tunnel release with additional procedures such as internal neurolysis, epineurotomy or tenosynovectomy.

4. Open carpal tunnel release using various incision techniques.

The non-surgical treatment includes:

1. Drugs: oral or local steroids, non-steroidal anti-inflammatory drugs (NSAIDs), diuretics, pyridoxine, etc.

2. Wrist splints.

3. Physical therapy, therapeutic exercises and manipulations. (ultrasound, laser therapy, yoga, and acupuncture, etc).

Research articles were excluded from review if they met the following criteria:

1. The study investigated the efficacy of two surgical interventions or two non-surgical managements.

2. The study did not provide data on intervention effectiveness).

3. The study published before 1970.

### Types of outcome measures

Primary outcome:

The primary outcome measure was patient self-reported functional and symptoms improvement at six months of follow-up. We selected this time point because most studies discussed the post operative status 6 months after the intervention.

Secondary outcomes:

1. Patient self-reported functional and symptoms improvement at three months of follow-up.

2. Patient self-reported functional and symptoms improvement at twelve months of follow-up.

3. Improvement of neurophysiological parameters.

4. Complications and side-effects.

### Data collection

Study authors (QS and JM)) independently performed the study selection, assessment of methodological quality and data abstraction. Structured data extraction forms were used to extract data on the characteristics of individual studies. Information was collected on participants (age, sex, diagnostic criteria used to confirm CTS, severity of symptoms, duration of symptoms, inclusion/exclusion criteria, trial setting, allocation procedure, blinding, number of participants or hands randomized), interventions (description of interventions, treatment length, number and explanation for any drop-outs) and outcome measures (description of measures used, continuous/dichotomous nature). We used the Cohen's (unweighted) kappa to assess the agreement between the two reviewers on study selection.

### Validity assessment

All the articles were assessed by two reviewers (QS, JM) using Jadad et al. scale [10] (see Additional File 2) and the Structured Effectiveness Quality Evaluation Scale (SEQES) (see Additional File 3) independently. All the disagreement was solved by consensus discussion.

Jadad et al. scale is used to assess the methodology quality of each study. There are 3 criteria for this scale and total score ranges from 0 to 5. We decided that the study was high quality if the cumulative score was 3 or more. To add additional detail on the quality of studies we also used the SEQES [11]. The scale has 24 items, scored 2, 1, or 0 based on congruence with specific descriptors. In this review, each study was ranked as low, moderate, or high quality based on the cumulative score (/48) using the following metric:

Low quality: scores 1-16

Moderate quality: scores 17-32

High quality: scores 33-48

### Data synthesis

Statistical analysis was performed using Review Manager (RevMan) version 5.0 [12]. Relative risks (RR) were calculated for dichotomous outcomes and weighted mean differences (WMD) for continuous outcomes. Studies were compared for heterogeneity using the Chi-square statistic (P-value < 0.05 considered statistically significant) and an I^2 ^test ( I^2 ^>50% considered substantial heterogeneity). A fixed- effects model was initially used in this systematic review. A random-effects model was applied if heterogeneity existed. We conducted a priori hypothesis to explain the heterogeneity that might exist between the studies. The potential sources were: difference in populations, severity of the disease, duration of the symptoms, intervention techniques, length of treatment and methodological quality.

## Results

### Description of included studies

There were 1333 articles identified from the literature research. Based on the abstracts review, only 10 articles potentially met the criteria for inclusion in this systematic review. Of these, 3 were excluded (Additional File 4) during evaluation of the full article based on the previously established exclusion criteria. Thus, seven primary studies [13-19] were included in the systematic review (Table 1). There was good agreement in the selection of trials (Cohen's unweighted kappa = 0.79).

**Table 1:** Summary of study Characteristics

ID	Authors	Year	Country	Design	Sample size	Inclusion and exclusion criteria	Study Quality (Jadad et al. Scores; SEQES)
1	Jarvik et al. [13]	2009	USA	RCT	**116** 57( OCTR or ECTR) 59( Multi-modality* )	1. Clinical diagnosis of CTS greater than 2 weeks 2. Confirmed by electrodiagnostic studies 3. In absence of electrodiagnostic criteria, positive in night pain and flick test 4. Excluded if previous treatment with CTS release surgery, severe thenar muscle atrophy	3/5 39/48

2	Elwakil et al. [14]	2007	Egypt	Comparative cohort study	**60** 30 (OCTR) 30 ( Laser )	Clinical diagnosis of CTS	1/5 29/48

3	Ucan et al. [15]	2006	Turkey	RCT	**57** 11( OCTR) 23 ( Splinting ) 23 ( Splinting + one dose steroid injection)	1. Mild to moderate clinical diagnosis of CTS greater than 6 months 2. Confirmed by electrodiagnostic studies 3. Excluded if advanced CTS, thenar atrophy or previous CTS treatment	2/5 36/48

4	Ly-Pen et al [16].	2005	Spain	RCT	**163** 80( OCTR) 83 (one or two-dose steroid injection )	1. Clinical diagnosis of CTS greater than 3 months 2. Confirmed by electrodiagnostic studies 3. Excluded if previous treatment with CTS release surgery, severe thenar muscle atrophy	3/5 35/48

5	Hui et al. [17]	2005	Hong Kong	RCT	**50** 25( OCTR) 25 ( One dose steroid injection )	1. Clinical diagnosis of CTS greater than 3 months but less than 1 year 2. Confirmed by electrodiagnostic studies 3. Excluded if severe thenar muscle atrophy, ulnar, radial neuropathy	3/5 38/48

6	Demirci et al. [18]	2002	Turkey	Comparative cohort study	**90** 44( OCTR) 46 ( Two-dose steroid injection )	1. Clinical diagnosis of CTS greater than 6 months 2. Confirmed by electrodiagnostic studies 3. Excluded if previous steroid injection, OCTR or distal radius fracture	0/5 31/48

7	Gerritsen et al [19]	2002	Netherlands	RCT	**176** 87( OCTR) 89 ( Splinting )	1. Clinical diagnosis of CTS 2. Confirmed by electrodiagnostic studies 3. Excluded if severe thenar muscle atrophy	3/5 40/48

RCT: randomized clinical trials

OCTR: standard open carpal tunnel release

ECTR: Endoscopic carpal tunnel release

We assessed seven studies including 5 RCTs and 2 controlled trials in this review. Overall, three studies compared surgery with steroid injection[16.17.18], two for surgery versus multi- modality [13,15] one for splinting [19] and one for laser [14]. There was homogeneity in entry criteria. The majority of patients enrolled in studies had clinical diagnosis of CTS confirmed by electrodiagnostic studies. Those had severe thenar muscle atrophy were excluded since these cases are not typically considered appropriate for conservative management.

The methodological features of each study are summarized in additional files 5 and 6. Totally four studies [13,16,17,19] rank high quality according to Jadad Scale. Because of lack of appropriate blinding, all the studies were rated as zero at the criteria of "double blinding". In Demirci et al. and Elwakil et al. articles, there were no adequate randomization performed so that both studies got zero in this criteria.

The quality of all studies ranges from 29 to 40/48 using SEQES. There are two high quality studies evaluated the multi-modality (SEQES = 35-39), two high quality studies and one moderate for the steroid (SEQES = 31-38), one high quality study for the splinting (SEQES = 40), one moderate for the laser (SEQES = 29). The common shortcomings of the studies were lack of blinding and inadequate randomization.

All studies concluded that both surgical and non-surgical intervention were beneficial to patients. However, there were no consistent outcome measures among identified studies. Patient self-reported scales, researcher-assessed subjective impairments, muscle strength and electrophysiological studies were commonly used in these studies. Five studies [13,15,16,18,19] employed patient self-administered functional and symptomatic scale questionnaires to evaluate the effect of the surgery. Although, these questionnaires varied across studies we were able to pool four studies that included scales for disease specific hand function and symptom to conduct a meta-analysis.

### Patient self-administered scales

One high quality [19] and two moderate quality studies [15,18] compared surgery and steroid injection and/or splinting by assessing outcomes using the Boston Questionnaire [20]. The Boston Questionnaire is a CTS specific tool for patient to self-report the symptom severity (11 items) and functional status (8 items). The over-all score is calculated as the mean of all items which is from 1 to 5. The higher the score is, the worse the symptom or function is. In previous studies [20,21], the validity and reliability of Boston Questionnaire has been tested. One high quality study [13] compared surgery and splinting with Carpal Tunnel Syndrome Assessment Questionnaire (CTSAQ) which is similar to the Boston Questionnaire. CTSAQ is modified from the Boston Questionnaire which includes 11 questions for symptom and 9 questions for function. Also, the reliability and responsiveness of CTSAQ has been verified [20,22]. Gerritsen's study [19] reported improvement score rather than ending point score in their outcome assessment. We included these results in our meta-analysis because they presented same direction of difference.

**Figure 1:**
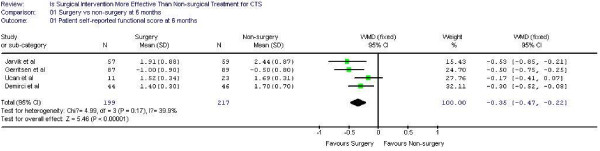
Patient self-reported functional improvement at 6 months

Figure 1,2 demonstrates the pooled functional and symptoms score at 6 months of follow up. We found that surgical was superior to the non surgical intervention at six months with the weighted mean difference 0.35 (95% CI 0.22, 0.47) for functional status and 0.43 (95% CI 0.29, 0.57) for symptom severity. Figure 3,4,5,6 presented the postoperative status score at 3 and 12 months. There were no statistically significant difference between surgery and non-surgery with regard to symptoms or functional score at 3 months, but there was a benefit in favor of surgery in terms of function and symptom relief at 12 months ( 0.35, 95% CI 0.15 to 0.55 and 0.37, 95% CI 0.19 to 0.56).

**Figure 2:**
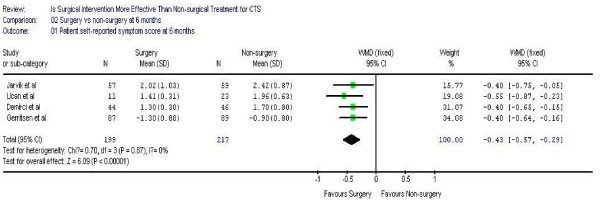
Patient self-reported symptom improvement at 6 months

**Figure 3:**
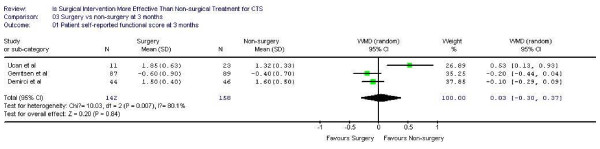
Patient self-reported functional improvement at 3 months

**Figure 4:**
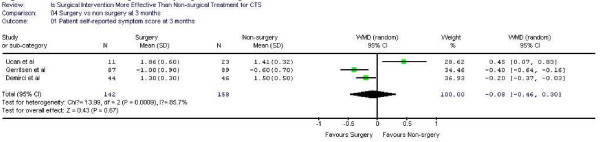
Patient self-reported symptom improvement at 3 months

**Figure 5:**
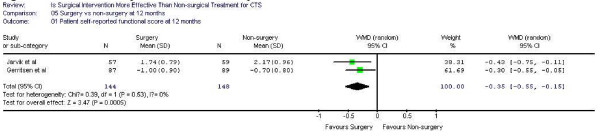
Patient self-reported functional improvement at 12 months

**Figure 6:**
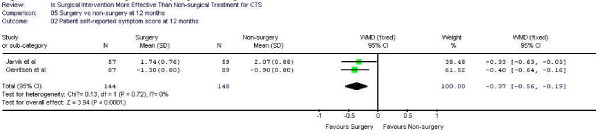
Patient self-reported symptom improvement at 12 months

### Electrophysiological studies

**Figure 7:**
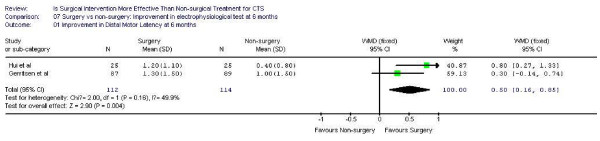
Improvement in distal motor latency at 6 months

**Figure 8:**
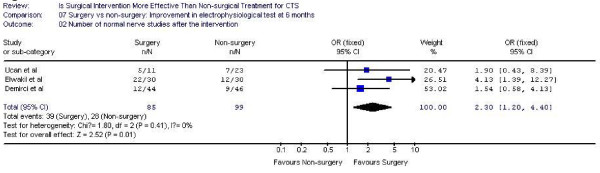
Number of normal nerve studies after intervention

Five studies [14,15,17-19]evaluated the electrophysiological improvement 6 months after the intervention. Two high quality trials [17,19] measured the median motor nerve distal latency while three moderate quality studies [14,15,18]assessed the number of normal nerve conduction tests at 6 months follow up. Surgery was found to be superior to conservative management regarding to the improvement of electrophysiological studies (Figure 7 and 8). For median motor nerve distal latency, the pooled effect size was 0.5 (95% CI 0.16, 0.85), indicating 50% more patients got normal distal latency after surgery. The relative risk of having normal nerve conduction tests after treatment was 2.3 (95% CI 1.2, 4.4), also favoring surgery.

### Complication and side effect

**Figure 9:**
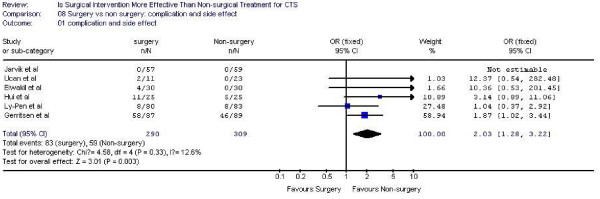
Complication and side effect

Six studies [13-17,19]reported complication and side effect of surgery and medication intervention ( Figure 9). A number of minor adverse effects were reported including: painful or hypertrophic scar, stiffness, swelling or discomfort of the wrist, most of them were resolved spontaneously in few weeks. Some authors [18] reported all complication regardless the severity while others only declared clinically important adverse events. This results in a large variation across studies in terms of complication rates. Overall, the pooled relative risk indicated a higher rate of complications in the surgical group (RR = 2.03, 95% CI 1.28 to 3.22). The most common complications reported in the surgical group were skin irritation and wound hematoma; while the complication reported with splinting was swelling of the wrist, hand and finger.

## Discussion

Despite, the limitation in the number of randomized controlled trials available in current literature, this systematic review was able to provide evidence that CTS symptoms improved in both interventions.

All the studies reported that both conservative managements (splinting, steroid and laser therapy) and surgery result in clinically significant improvement in symptoms. Some authors [13,15,17-19] concluded that surgical decompression produces long-term systematic improvement compared with the non-surgical intervention. We found that the positive impact of conservative management plateaus within 3 months whereas, the clinical effect of surgical intervention up until 12^th ^months after the treatment. The relative advantage of surgery at 6 months (WMD = 0.35) indicated that patient with surgical release had approximately 0.35 points lower functional scores than those receiving conservative intervention.. Although there was a similar trend at 12 months, no further improvement was observed at 12 months of follow-up. Thus, the current treatment approach of providing a conservative management as a front-line treatment in mild to moderate cases before considering surgery is justified.

However, surgery was superior to the non surgical intervention regarding the improvement of electrophysiological study. The relative advantage of surgery (RR = 2.3) indicated that approximately twice as many patients achieve better outcomes with surgery. This is important information for patient who fails conservative management to understand when deciding whether they should consent to surgery.

Prognosis was not addressed in these study trials but others have indicated that patients presenting with higher symptom severity scores and those not responding within the first six weeks are more likely to proceed to surgery following conservative management [23]. Given that the size of the treatment advantage for surgical management is relatively small, and that improvements are noted with both conservative and surgical approaches the evidence does not support proceeding directly to surgery. The presenting symptoms/nerve damage, response/relief after conservative management, comorbid issues and patient circumstances/preferences will determine the optimal decision about surgery. There are potential complications that patients must consider, in particular for surgical management or steroid injection. Given the huge variation of how complications are defined, this systematic review was not well positioned to determine accurate rates of these complications.

Our review indicates substantial heterogeneity in effects between studies. This may have resulted from variations between the studies in terms of intervention techniques, length of treatment, methodological quality, etc. For example, all the patients in splinting group received 6 weeks treatment in Gerritsen study [19] while patients in Ucan study [15] used the splinting for 3 months. For this reason future systematic reviews that included larger numbers of studies might be useful to differentiate subgroups who would benefit most from conservative versus surgical management or factors associated with successful treatment in either treatment arm.

Critical appraisal of trials involving surgery, or hands-on interventions within the scope of conservative management have some inherent challenges in blinding that affect their scores on most critical appraisal instruments. While the Jadad scale is commonly used, others have pointed out its lack of reliability and validity with respect to surgery and rehabilitation research [24,25]. For this reason we used a 24-item structured evaluation instrument [26] that has been used in other hand surgery/therapy systematic reviews [27,28]. This instrument also provides extra credit for blinding, but has an intermediary score for cases where blinding is not possible. In addition, because it addresses a variety of aspects of study in addition to blinding there is an opportunity for well-designed surgery trials to be favorably rated despite a lack of blinding.

One limitation of this systematic review is only studies written in English were included, which might introduce a publication bias. However, one recent assessment reported that non-English papers are likely to be of low quality and could result in bias into a review [29].

## Further Research

We observed a small to moderate incremental benefit in surgical group for patients with carpal tunnel syndrome. However, given that conservative management is effective in relieving symptoms and can circumvent the need for surgery in a certain proportion of cases it remains a justified first line treatment. Therefore, we do not see a need for further trials comparing conservative management versus surgical management but rather a need for better prognostic studies that would identify the characteristics of patients most likely to respond to each type of intervention. This would form a basis for clinical prediction rules and clearer criteria for which patients should be fast tracked to surgery and how long conservative management should be sustained before making decisions about transitioning into a surgical procedure.

## Conclusion

This systematic review presents that both surgical and conservative interventions are beneficial in the management of carpal tunnel syndrome. Surgical treatment provides a better outcome up to twelve months in terms of symptoms and restoration of normal nerve conductions test results; but has higher complication risk. Most complications of CTS interventions are mild. Since conservative interventions are beneficial for a substantial proportion of patients and effects plateau within three months the traditional approach to use a trial of conservative management in patients with mild and moderate or transient CTS is supported by evidence.

## Competing interests

The authors declare that they have no competing interests.

## Authors' contributions

QS Participated in the design of the study, performed the statistical analysis and drafted the manuscript. JM participated in its design and coordination and helped to draft the manuscript. All authors read and approved the final manuscript.

## Supplementary Material

Additional file 1 **Search strategy of systematic review**; Search strategy for 4 databases Click here for file

Additional file 2 **Jadad et al. Scale**. description of Jadad scale Click here for file

Additional file 3 **Structured Effectiveness Quality Evaluation Scale (SEQES)**. description of SEQES Click here for file

Additional file 4 **Excluded studies**. summary of excluded studies ( study identity, reason for exclusion) Click here for file

Additional file 5 **Study Quality (Jadad et al. scores) for 7 included articles**. summary of Jadad score in included studies Click here for file

Additional file 6 **Appendix 6 Study Quality (SEQES scores) for 7 included articles**. summary of SEQES score in included studies Click here for file
