# Supplementary material for: Is surgical intervention more effective than non-surgical treatment for carpal tunnel syndrome? a systematic review
Source: J Orthop Surg Res. 2011 Apr 11;6:17. doi: 10.1186/1749-799X-6-17 (PMC3080334; doi:10.1186/1749-799X-6-17)
Supplement: Additional file 1 — Search strategy of systematic review; Search strategy for 4 databases [file 1749-799X-6-17-S1.DOC]

### Additional Files 1

**Ovid MEDLINE Search Strategy**

**Ovid MEDLINE**

1 carpal tunnel syndrome.mp. or Carpal Tunnel Syndrome/

2 (carp$ tunn$ or tunn$ syndrom$or carp$syndrom$).mp. [mp=title, original title, abstract, name of substance word, subject heading word]

3 median nerve entrapment.mp.

4 nerve compression syndromes.mp. or Nerve Compression Syndromes/

5 1 or 2 or 3 or 4

6 controlled trial.mp.

7 clinical trial.mp. or Clinical Trial/

8 6 or 7

9 Comparative Study/

10 prospective studies.mp. or Prospective Studies/

11 follow up studies.mp. or Follow-Up Studies/

12 9 or 10 or 11

13 8 and 12

14 5 and 13

**Ovid EMBASE Search Strategy**

**Ovid EMBASE**

1 (carpal tunnel syndrome or Carpal Tunnel Syndrome).mp. [mp=title, abstract, subject headings, heading word, drug trade name, original title, device manufacturer, drug manufacturer name]

2 (carp$ tunn$ or tunn$ syndrom$ or carp$syndrom$).mp. [mp=title, abstract, subject headings, heading word, drug trade name, original title, device manufacturer, drug manufacturer name]

3 (nerve compression syndromes or Nerve Compression Syndromes or nerve entrapment).mp. [mp=title, abstract, subject headings, heading word, drug trade name, original title, device manufacturer, drug manufacturer name]

4 1 or 2 or 3

5 Controlled Study/

6 (clinical trial or Clinical Trial).mp. [mp=title, abstract, subject headings, heading word, drug trade name, original title, device manufacturer, drug manufacturer name]

7 5 or 6

8 4 and 7

9 (surgical approach or carpal tecnique).mp. [mp=title, abstract, subject headings, heading word, drug trade name, original title, device manufacturer, drug manufacturer name]

10 (surgery or surgical or operation or reconstruct$).mp. [mp=title, abstract, subject headings, heading word, drug trade name, original title, device manufacturer, drug manufacturer name]

11 (epineurotomy or carpal tunnel release or epineurotomy).mp. [mp=title, abstract, subject headings, heading word, drug trade name, original title, device manufacturer, drug manufacturer name]

12 9 or 10 or 11

13 8 and 12

**The Search Strategy of Cochrane Central Register for Controlled Trials**

There are 311 results out of 522340 records for: "median nerve entrapment” or “carpal tunnel syndrome” or “entrapment neuropathy” in Title, Abstract or Keywords.

**The search strategy for PEDro**

“carpal tunnel syndrome” OR “median nerve entrapment” OR “entrapment neuropathy”
