# Supplementary material for: Is surgical intervention more effective than non-surgical treatment for carpal tunnel syndrome? a systematic review
Source: J Orthop Surg Res. 2011 Apr 11;6:17. doi: 10.1186/1749-799X-6-17 (PMC3080334; doi:10.1186/1749-799X-6-17)
Supplement: Additional file 2 — Jadad et al. Scale. description of Jadad scale [file 1749-799X-6-17-S2.DOC]

##### Additional file 2

Jadad et al. Scale*

Please read the article and try to answer the following questions (see attached instructions):

1. Was the study described as randomized (this includes the use of words such as randomly, random, and randomization)?

2. Was the study described as double blind?

3. Was there a description of withdrawals and dropouts?

Scoring the items:

Either give a score of 1 point for each “yes” or 0 points for each “no.” There are no in-between marks.

Give 1 additional point if: For question 1, the method to generate the sequence of randomization was described and it was appropriate (table of random numbers, computer generated, etc.)

And/or: If for question 2 the method of double blinding was described and it was appropriate (identical

placebo, active placebo, dummy, etc.)

Deduct 1 point if: For question 1, the method to generate the sequence of randomization was described and it was inappropriate (patients were allocated alternately, or according to date of birth, hospital number, etc.)

And/or For question 2, the study was described as double blind but the method of blinding was inappropriate (e.g., comparison of tablet versus injection with no double dummy)

Guidelines for assessment

1. Randomization

A method to generate the sequence of randomization will be regarded as appropriate if it allowed each study participant to have the same chance of receiving each intervention and the investigators could not predict which treatment was next. Methods of allocation using date of birth, date of admission, hospital numbers, or alternation should be not regarded as appropriate.

2. Double blinding

A study must be regarded as double blind if the phrase “double blind” is used. The method will be regarded as appropriate if it is stated that neither the person doing the assessments nor the study participant could identify the intervention being assessed, or if in the absence of such a statement the use of active placebos, identical placebos, or dummies is mentioned.

3. Withdrawals and dropouts

Participants who were included in the study but did not complete the observation period or who were not included in the analysis must be described. The number and the reasons for withdrawal in each group must be stated. If there were no withdrawals, it should be stated in the article. If there is no statement on withdrawals, this item must be given no points.

*** From Jadad, A. R., Moore, A., Carroll, D., et al. Assessing the quality of reports of randomized clinical trials: Is blinding necessary? *Control. Clin. Trials* 17: 1, 1996.**
