# Supplementary material for: Is surgical intervention more effective than non-surgical treatment for carpal tunnel syndrome? a systematic review
Source: J Orthop Surg Res. 2011 Apr 11;6:17. doi: 10.1186/1749-799X-6-17 (PMC3080334; doi:10.1186/1749-799X-6-17)
Supplement: Additional file 3 — Structured Effectiveness Quality Evaluation Scale (SEQES). description of SEQES [file 1749-799X-6-17-S3.DOC]

##### Additional file 3

Structured Effectiveness Quality Evaluation Scale (SEQES)*

# Evaluation Criteria Score Study question

**2 1 0**

**Study question**

1. Was the relevant background work cited to establish a foundation for the research question?

**Study design**

2. Was a comparison group used?

3. Was patient status at more than one time point considered?

4. Was data collection performed prospectively?

5. Were patients randomized to groups?

6. Were patients blinded to the extent possible?

7. Were treatment providers blinded to the extent possible?

8. Was an independent evaluator used to administer outcome measures?

**Subjects**

9. Did sampling procedures minimize sample/selection biases?

10. Were inclusion/exclusion criteria defined?

11. Was an appropriate enrollment contained?

12. Was appropriate retention/follow-up obtained?

**Intervention**

13. Was the intervention applied according to established principles?

14. Were biases due to the treatment provider minimized (ie attention, training)?

15. Was the intervention compared to appropriate comparator?

**Outcomes**

16. Was an appropriate primary outcome defined?

17. Were appropriate secondary outcomes considered?

18. Was an appropriate follow-up period incorporated?

**Analysis**

19. Was an appropriate statistical test(s) performed to indicate differences related to the intervention?

20. Was it established that the study had significant power to identify treatment effects?

21. Was the size and significance of the effects reported?

22. Were missing data accounted for and considered in analyses?

23. Were clinical and practical significance considered in interpreting results?

**Recommendations**

24. Were the conclusions/clinical recommendations supported by the study objectives, analysis, and results?

**Total quality score (sum of above)=**

*Joy MacDermid, 2003.
