# Supplementary material for: Is surgical intervention more effective than non-surgical treatment for carpal tunnel syndrome? a systematic review
Source: J Orthop Surg Res. 2011 Apr 11;6:17. doi: 10.1186/1749-799X-6-17 (PMC3080334; doi:10.1186/1749-799X-6-17)
Supplement: Additional file 4 — Excluded studies. summary of excluded studies ( study identity, reason for exclusion) [file 1749-799X-6-17-S4.DOC]

##### Additional file 4

**Excluded studies**

| Author | Year | Title | Reason for exclusion |
| --- | --- | --- | --- |
| Korthals-de Bos et al. | 2006 | Surgery is more cost-effective than splinting for carpal tunnel syndrome in the Netherlands: results of an economic evaluation alongside a randomized controlled trial. | A cost-effectiveness analysis of Gerritsen study^18^ |
| Ettema et al. | 2006 | Surgery versus conservative therapy in carpal tunnel syndrome in people aged 70 years and older | Retrospective study. |
| Martin et al. | 2005 | Randomized clinical trial of surgery versus conservative therapy for carpal tunnel syndrome | No result reported |

1
