# Supplementary material for: Is surgical intervention more effective than non-surgical treatment for carpal tunnel syndrome? a systematic review
Source: J Orthop Surg Res. 2011 Apr 11;6:17. doi: 10.1186/1749-799X-6-17 (PMC3080334; doi:10.1186/1749-799X-6-17)
Supplement: Additional file 5 — Study Quality (Jadad et al. scores) for 7 included articles. summary of Jadad score in included studies [file 1749-799X-6-17-S5.DOC]

Additional file 5: Study Quality (Jadad et al. scores) for 7 included articles

| Study | 1 | 2 | 3 | Quality Score |
| --- | --- | --- | --- | --- |
| Javik et al 2009^11^ | 2 | 0 | 1 | 3 |
| Elwakil et al 2007^12^ | 0 | 0 | 1 | 1 |
| Ucan et al 2006^13^ | 1 | 0 | 1 | 2 |
| Ly-Pen et al. 2005^14^ | 2 | 0 | 1 | 3 |
| Hui et al. 2005^15^ | 2 | 0 | 1 | 3 |
| Demirci et al 2002^16^ | 0 | 0 | 0 | 0 |
| Gerritsen et al 2002^17^ | 2 | 0 | 1 | 3 |

1: Randomization

2: Double blinding

3: Withdrawals and dropout
