# Supplementary material for: Is surgical intervention more effective than non-surgical treatment for carpal tunnel syndrome? a systematic review
Source: J Orthop Surg Res. 2011 Apr 11;6:17. doi: 10.1186/1749-799X-6-17 (PMC3080334; doi:10.1186/1749-799X-6-17)
Supplement: Additional file 6 — Appendix 6 Study Quality (SEQES scores) for 7 included articles. summary of SEQES score in included studies [file 1749-799X-6-17-S6.DOC]

Additional file 6 Study Quality (SEQES scores) for the 7 included articles

|  | SEQES evaluation criteria | | | | | | | | | | | | | | | | | | | | | | | | |
| --- | --- | --- | --- | --- | --- | --- | --- | --- | --- | --- | --- | --- | --- | --- | --- | --- | --- | --- | --- | --- | --- | --- | --- | --- | --- |
| Citations | 1 | 2 | 3 | 4 | 5 | 6 | 7 | 8 | 9 | 10 | 11 | 12 | 13 | 14 | 15 | 16 | 17 | 18 | 19 | 20 | 21 | 22 | 23 | 24 | total |
| Javik et al 2009^11^ | 2 | 2 | 2 | 2 | 2 | 0 | 1 | 1 | 1 | 2 | 2 | 0 | 2 | 1 | 2 | 2 | 2 | 2 | 1 | 2 | 2 | 2 | 2 | 2 | 39 |
| Elwakil et al 2007^12^ | 1 | 2 | 2 | 2 | 0 | 0 | 1 | 1 | 0 | 1 | 0 | 2 | 2 | 1 | 2 | 2 | 0 | 2 | 2 | 2 | 0 | 2 | 1 | 1 | 29 |
| Ucan et al 2006^13^ | 2 | 2 | 2 | 2 | 1 | 0 | 0 | 0 | 0 | 2 | 2 | 2 | 2 | 2 | 2 | 2 | 1 | 2 | 2 | 2 | 1 | 1 | 2 | 1 | 35 |
| Ly-Pen et al. 2005^18^ | 2 | 2 | 2 | 1 | 2 | 0 | 1 | 0 | 0 | 2 | 2 | 2 | 2 | 2 | 2 | 2 | 1 | 2 | 2 | 2 | 1 | 0 | 2 | 1 | 35 |
| Hui et al. 2005^19^ | 2 | 2 | 2 | 2 | 2 | 0 | 1 | 1 | 1 | 2 | 2 | 2 | 2 | 2 | 2 | 2 | 2 | 2 | 2 | 2 | 2 | 2 | 2 | 2 | 38 |
| Demirci et al. 2002^17^ | 2 | 2 | 2 | 2 | 0 | 0 | 0 | 0 | 1 | 2 | 0 | 2 | 2 | 0 | 2 | 2 | 0 | 2 | 2 | 2 | 2 | 1 | 2 | 1 | 31 |
| Gerritsen et al 2002^15^ | 2 | 2 | 2 | 2 | 2 | 0 | 0 | 2 | 1 | 2 | 2 | 1 | 2 | 0 | 2 | 2 | 2 | 2 | 2 | 2 | 2 | 2 | 2 | 2 | 40 |

0: the criteria has not been met,

1: indicates that the criterion has been partially met

2: indicates that the criterion has been fully met
